# VGLL4 interacts with STAT3 to function as a tumor suppressor in triple-negative breast cancer

**DOI:** 10.1038/s12276-019-0338-8

**Published:** 2019-11-20

**Authors:** Hongming Song, Qifeng Luo, Xiaochong Deng, Changle Ji, Dengfeng Li, Amik Munankarmy, Wei Jian, Junyong Zhao, Lin Fang

**Affiliations:** 1 Department of Breast and Thyroid Surgery, Shanghai Tenth People’s Hospital, School of Medicine, Tongji University, 200072 Shanghai, China; 2 Breast Disease Center, The Affiliated Hospital of Qingdao University, 59 Haier Road, Qingdao, 266000 Shandong China

**Keywords:** Cell signalling, Breast cancer

## Abstract

Triple-negative breast cancer (TNBC) is an aggressive malignancy with a poor prognosis, and there are no effective molecular-targeted drugs for TNBC patients in clinical practice. The JAK-STAT pathway is implicated in tumorigenesis and the progression of various cancers. In this study, the results demonstrated that VGLL4 is expressed at low levels in both TNBC specimens and cell lines and that VGLL4 expression is negatively correlated with Ki67 expression and tumor size in TNBC patients. VGLL4 knockdown can promote the growth of TNBC cells, while VGLL4 overexpression significantly suppresses the growth of TNBC cells in vitro. More importantly, VGLL4 significantly inhibits tumor progression in a nude mouse model. In addition, VGLL4 is a direct target of miR-454, and the upregulation of miR-454 decreases VGLL4 expression and promotes the cell growth of TNBC cells. Furthermore, we also demonstrated that VGLL4 interacts with STAT3, the core component of the JAK-STAT pathway, leading to the inactivation of STAT3 and the inhibition of STAT3 downstream transcription. Collectively, these findings indicate that VGLL4 expression is negatively associated with poor prognosis in TNBC patients. High expression of miR-454 may be one of the causes of the downregulation of VGLL4 in TNBC, and VGLL4 acts as a tumor suppressor in TNBC by interacting with STAT3 and subsequently suppresses the STAT3 signaling axis, providing potential biomarkers and therapeutic approaches for this fatal disease.

## Breast cancer: New targets for treating aggressive disease

Targeting the regulation or activity of a tumor suppressor protein that is deactivated in triple-negative breast cancer (TNBC) holds promise for the treatment of this highly aggressive form of cancer. Lin Fang and colleagues at Tongji University in Shanghai, China, showed that low levels of the protein vestigial-like family member 4 (VGLL4) promoted TNBC cell proliferation and migration, whereas overexpression of VGLL4 prevented the growth of these cells in mice. VGLL4 acts as a tumor suppressor by interfering with a signaling pathway that drives tumor growth. Furthermore, they found that VGLL4 expression is regulated by a small RNA molecule that is highly expressed in TNBC patients with poor prospects of survival. These findings provide new insights into the mechanisms underlying TNBC and highlight potential strategies for the development of targeted therapies.

## Introduction

Triple-negative breast cancers (TNBCs) represent approximately 10–15% of all breast cancers and are a highly aggressive subtype of tumors that lack estrogen receptor (ER), progesterone receptor (PR) and HER2 gene amplification^1,2^. Given the specific receptor status in TNBC, there are no clearly defined TNBC-specific therapeutic targets, and patients with TNBC do not benefit from endocrine therapy or anti-HER2 therapy. Systematic chemotherapy is currently the primary modality used for the treatment of TNBC patients. However, long-term use may result in chemotherapeutic resistance, leading to tumor recurrence and metastasis.

In recent years, a few molecules have been identified as biomarkers and therapeutic targets for TNBC. For example, pembrolizumab, a PD-L1 antagonistic monoclonal antibody, as a first-line therapy for patients with PD-L1-positive mTNBC, exerts robust anti-tumor activity^3^; in addition, the poly (ADP-ribose) polymerase (PARP) inhibitor olaparib, may be effective against BRCA mutated-mTNBC^4^, and the combination of olaparib and chemotherapy appears to be a promising approach for increasing treatment efficacy in BRCA mutated-TNBC^5–7^. These findings provide us with new therapeutic horizons beyond conventional chemotherapy for TNBC. Therefore, much effort is still needed to find novel therapeutic targets for the treatment of TNBC.

Vestigial-like family member 4 (VGLL4) is a transcriptional cofactor of the VGLL family. In Drosophila, this gene is homologous to the vestigial (VG) gene, and the loss of its expression can hinder wing formation by interfering with cell proliferation in the wing imaginal disc^8^. The role of VGLL4 varies widely in different cells and tissues. Overexpression of VGLL4 enhances the colony formation ability of human embryonic stem cells. Abnormal expression of VGLL4 is associated with the development of various cancers, such as lung cancer, gastric cancer and pancreatic cancer^9–12^. Unlike other VGLL family proteins, VGLL4 has two TDU motifs. It uses these motifs to interact with TEA domain transcription factors (TEADs), thereby executing its biological function. VGLL4 acts as a tumor suppressor in lung cancer cells, and it competes with YAP to bind to TEAD4 and inhibits the downstream transcription of TEAD4^12^. However, the role of VGLL4 in TNBC is still unclear. Its ability to interact through its TDU motifs needs further elucidation.

Biotinylated proteins can be powerful tools to assess protein–protein interactions. Biotin-protein ligase (BirA enzyme) has the ability to biotinylate proteins and has been widely used as an important reagent for the study of interactions between proteins^13,14^. Biotinylated proteins can be detected by streptavidin. Some scholars have modified the BirA enzyme called BirA*, which is capable of biotinylating its surrounding proteins without recognizing specific amino acids^15,16^. Biotinylation site identification technology (BioSITe) was modified on the basis of traditional biotinylated protein identification (BioID) technology^17^. It is a good method to explore candidate proteins that interact with the TDU domain of the VGLL4 protein. In addition, microRNAs (miRNAs) are a class of highly conserved endogenous short noncoding RNAs with a length of 18–24 nucleotides that function as negative regulators of gene expression by binding to the 3′-untranslated region (3′-UTR) of their target gene mRNAs. Abnormal expression of miRNAs is associated with many cancers, including breast cancer^18–20^. To explore the functional miRNAs that target VGLL4, it is helpful to understand the expression and function of VGLL4 in TNBC.

In the present study, we demonstrated that VGLL4 expression is lower in TNBC samples and cell lines and that VGLL4 expression is negatively correlated with Ki67 expression in TNBC. In addition, we showed that VGLL4 knockdown promotes proliferation and migration and inhibits apoptosis in TNBC cells. VGLL4 overexpression acts as a tumor suppressor in TNBC cells both in vitro and in vivo. In addition, miR-454 directly targets the VGLL4 3′UTR, leading to VGLL4 inactivation and the promotion of TNBC cell growth. This may also be one of the reasons for the trend of low expression of VGLL4 in TNBC. More importantly, we observed that VGLL4 inactivates STAT3 by interacting with the STAT3 protein, leading to the downregulation of transcriptional products of STAT3, such as Cyclin D1 and Bcl-2.

## Materials and methods

### Patient samples

All patients gave their informed consent prior to their inclusion in the study, and the study protocols were approved by the Institutional Ethics Committees of the Hospital (the approval number: SHSY-IEC-KY-4.0/17-83/01). Samples from 35 cases of TNBC and matched normal adjacent tissues were obtained from the Department of Breast and Thyroid Surgery of Shanghai Tenth People’s Hospital. The samples were snap-frozen in liquid nitrogen immediately after surgery and stored at −80 °C.

### Cell culture and transfection

The breast cancer cell lines BT-549, MDA-MB-231, MDA-MB-468, HCC1937, MCF-7, T-47D, MDA-MB-436 were obtained from the cell bank of the Chinese Academy of Science (Shanghai, China), where they were characterized based on mycoplasma detection, DNA fingerprinting, isozyme detection, and determination of cell viability. The human normal breast cell line MCF-10A was purchased from Zhongqiaoxinzhou Biotech (Shanghai, China) and tested and authenticated prior to use. The BT-549 cells were cultured in RPMI 1640 (Gibco, USA) supplemented with 10% fetal bovine serum (FBS) (Gibco, USA) and 1% penicillin-streptomycin (PS, 100 μg/ml) (Enpromise, Hangzhou, China). The MCF10A cells were cultured in DMEM/F12 (Invitrogen, USA) supplemented with 5% horse serum (Invitrogen, USA), 1% penicillin-streptomycin (PS, 100 μg/ml), 20 ng/ml EGF, 0.5 mg/ml hydrocortisone, and 10 µg/ml insulin. The other cell lines were cultured in DMEM (Gibco, USA) containing 10% FBS and 1% PS. All cell lines were cultured in a 5% CO_2_ incubator at 37 °C.

The cells were plated into 6-well plates at a density of 1.0 × 10^5^ cells per well. When the cells reached 30–40% confluence, miR-454/miR-NC, anti-miR-454/anti-miR-NC (RiboBio, China), and si-VGLL4/si-NC (Sangon Biotech, China) were transiently transfected at a final concentration of 100 nmol/l into MDA-MB-231, HCC1937 or BT-549 cells using Lipofectamine 2000 (Invitrogen, USA). The cells were harvested 48 h posttransfection to assess transfection efficiency and for other follow-up experiments. For essential information on the synthetic sequences, see Supplementary Table 1.

### DNA expression plasmids and constructs

The pLenO-GTP-VGLL4-Flag vector construction and VGLL4 lentiviral particles were provided by Shanghai Lingke Biotechnology Co., Ltd (Shanghai, China). After infecting cells with VGLL4 lentiviral particles, MDA-MB-231 cells with stable VGLL4 stable overexpression were obtained by puromycin selection (final concentration of 1 µg/ml). To generate a BirA*-HA-tagged protein, the specific DNA was cloned into the pBABE-puro-BirA*-HA expression vector by Gibson Assembly® Master Mix (New England Biolabs, USA). MCF-10A cells with stable VGLL4 knockin were selected using puromycin.

### RNA extraction and quantitative reverse transcription PCR

Total RNA was extracted with Trizol reagent (Invitrogen), and 1 μg of total RNA was reverse transcribed into cDNA by using a PrimeScript™ RT-PCR kit (Takara) in accordance with the manufacturer’s instructions. Quantitative real-time PCR (qPCR) analysis was performed in triplicate using 10 ng of cDNA and SYBR® FAST qPCR Master Mix kit (Invitrogen, Burlington, ON, Canada) on an ABI PRISM 7500 series real-time PCR system (Life Technologies, Grand Island, NY, USA). The cycle conditions were as follows: 95 °C for 3 min, followed by 40 cycles of 95 °C for 3 s and 60 °C for 30 s. mRNA and miRNA expression was assessed by evaluating threshold cycle (CT) values. The expression of the indicated mRNA or miRNA was quantified by the 2^−ΔΔCt^ method. β-actin was utilized as an internal control gene to normalize gene expression. U6 RNA was used to normalize miRNA expression. The primers used in this study are listed in Supplementary Table 1.

### Western blot analysis

Cell or tissue samples were lysed for 30 min on ice with RIPA buffer (Beyotime, Shanghai, China), and soluble proteins were recovered from the supernatant following a 30-min centrifugation (12,000 rpm) at 4 °C. Western blot analysis was performed as previously described^21^.

### 3-(4,5-dimethylthiazol-2-yl)-2, 5-diphenyl-tetrazolium bromide (MTT) cell proliferation assay

MTT assays were performed to measure cell proliferation. MDA-MB-231 and HCC1937 cells were plated in 200 μl of culture medium in 96-well plates at a density of 500 cells per well. Briefly, 20 μl of MTT solution (5 mg/mL) (Sigma-Aldrich, St. Louis, MO, USA) was added to each well, and the plate was incubated for 4 h at 37 °C with 5% CO_2_. The media was then removed, and 150 μl of DMSO was added to each well. The absorbance was measured at an optical density (OD) of 490 nm using a microplate reader (BioTek, Winooski, VT, USA).

### Colony formation assay

The cells were plated in 6-well plates at a density of 5 × 10^2^ cells per well and incubated at 37 °C for 7–10 days. Before harvesting, the cells were thoroughly checked under a microscope to ensure proper confluence and health status. The cells were gently washed twice with PBS, fixed with 4% paraformaldehyde (PFA) for 15 min and then stained with 0.1% crystal violet solution for 20 min. Then, the plate was slowly washed three times with water. The plates were photographed using a digital camera, and colonies of more than 50 cells were counted.

### Cell migration assays

For the migration assays, 24-well Boyden chambers (Corning Incorporated, Corning, NY, USA) were used (8.0-mm pore size, Falcon) without Matrigel-coated filters for migration. Cells (5 × 10^4^) were seeded in the upper chambers and then cultured at 37 °C (16 h for MDA-MB-231 cells, 24 h for BT-549 cells). The migrated cells were fixed in 4% PFA and stained with 0.5% crystal violet (Sigma) after the cells that remained on the upper side of the filter were removed. Cells from five randomly selected microscopic fields per well were counted.

### Flow cytometry assay

For analysis of the cell cycle, cells were fixed in 70% ethanol overnight at 4 °C. The cells were washed twice with PBS and stained with propidium iodide (PI) for 30 min at room temperature. For assaying cell apoptosis, 24 h after plating in cell culture medium or transfection, cells were treated with 10 mmol/L 5-fluoro-2,4(1 H,3 H)-pyrimidinedione (5-FU) (Xudonghaipu, Shanghai) to induce apoptosis for 36 h. The cells were then collected and washed with cold PBS. Subsequently, apoptosis was detected using the Annexin V-FITC Apoptosis Detection kit (BD Biosciences, San Diego, CA, USA) according to the manufacturer’s protocol. The cell-cycle distribution and the rate of apoptosis were determined using BD FACSAria III (BD Biosciences, San Jose, CA, USA).

### Animal studies

All research involving animals complied with protocols that were approved by the Shanghai Institution Animal Feed and Use Committee (study protocol number: SHDSYY-0221). MDA-MB-231 cells with stable overexpression of VGLL4 or its negative control (NC) were used for animal experiments in vivo. Cell suspensions (3 × 10^6^ MDA-MB-231 cells with overexpression of VGLL4 or VGLL4-NC per mouse) were injected into the mammary fat pads of 6-week-old female mice. Six weeks later, the tumor sizes were determined every 3 days. Five weeks later, the mice were sacrificed, and the tumors were harvested, weighed, and photographed.

### Immunoprecipitation (IP) assay

Cells were lysed in RIPA buffer (Beyotime), and an appropriate amount of the protein lysates was mixed with 30 µl of protein A/G plus-agarose (Santa Cruz, Dallas, TX, USA) after protein quantification. Primary antibody was added to the mixture and incubated at 4 °C overnight on a rotating device. Then, the precipitated proteins were mixed with loading buffer for western blot analysis.

### Immunohistochemical staining

Immunohistochemical staining was performed on formalin-fixed, paraffin-embedded breast cancer and paracancerous tissues. Briefly, paraffin-embedded slides were rehydrated, treated with hydrogen peroxide to block endogenous peroxidase activity, and then washed with PBS buffer. The slides were then blocked with goat serum before diluted primary antibodies were added for protein binding and incubated at 4 °C overnight. Then, the slides were incubated with biotinylated secondary antibodies and with streptavidin–horseradish peroxidase complex using the Biotin-Streptavidin HRP Detection Kit (SP-9000, ZSGB-BIO, Beijing, China). The slides were then treated with the DAB substrate kit (Dako) according to the manufacturer’s instructions. The slides were visualized under the microscope, and five views per slide were selected randomly for evaluation.

### Dual-luciferase reporter assay

Wild-type or mutant human VGLL4 3′-UTR segments (See Supplementary Table 1 for sequences) were cloned into a luciferase reporter vector (psiCHECK-2, obtained from IBSBIO, Shanghai, China). According to the manufacturer’s protocol, MDA-MB-231 cells were cotransfected with 40 ng of VGLL4 3′-UTR luciferase construct and miR-454 mimic or NC-mimic (Ruibo, 50 nM) in 48-well plates using Lipofectamine 2000 (Invitrogen). Cells were collected 24 h after transfection, and the luciferase activity was assessed using the Dual-Luciferase Reporter Assay system (Promega, Madison, WI, USA). Firefly luciferase activity was normalized to Renilla luciferase activity, with the results are presented as the ratio of FL activity to RL activity (FL/RL).

### EdU staining proliferation assay

EdU solution was diluted with cell culture medium (1000:1) to prepare 50 μM EdU medium; 300 μl of medium containing 50 μM EdU solution was added to each well of a 6-well plate, and the cells were cultured for 2 h. The cells were fixed in PBS containing 4% paraformaldehyde for 30 min at room temperature and then stained with Apollo® staining reaction solution and Hoechst 33342 reaction solution for 30 min at room temperature. Next, the wells were washed with PBS 2 to 3 times. Finally, five fields of view from each well were randomly selected for image acquisition and analysis under a fluorescence microscope.

### Stable isotope labeling with amino acid in cell cultures (SILAC) and biotinylation site identification technology (BioSITe)

We constructed a full-length VGLL4 amino acid sequence (VGLL4-BirA*-HA), a C-terminal VGLL4 amino acid sequence comprising the first TDU domain (C-VGLL4-BirA*-HA), and an N-terminal VGLL4 amino acid sequence comprising the second TDU domain (N-VGLL4-BirA*-HA). The above sequence ends fused with the BirA protein. SILAC was used to label the cells and divide them into different groups. VGLL4-BirA*-HA was cultured with normal medium (light group), and N-VGLL4-BirA*-HA (medium group) and C-VGLL4-BirA*-HA (heavy group) were cultured in K4R6 (^13^C_6_^14^N_4_-arginine + ^2^H_4_-lysine)- and K8R10 (^13^C_6_^15^N_4_-arginine + ^13^C_6_^15^N_2_-lysine)-labeled medium, respectively. After 5–6 passages, the labeling efficiency was checked before BioSITe. For BioSITe, cells were passaged overnight at 50% density. The next day, biotin (Sigma-Aldrich, USA) was added to label the cells for 12 h. Then, the proteins were collected and quantified. Part of the protein sample was taken to test the biotin labeling efficiency. After confirming the labeling efficiency, 5 mg of protein from each group was mixed and used for subsequent experiments. Proteins were treated with dithiothreitol (Sigma-Aldrich, USA) and iodoacetamide (Sigma-Aldrich, USA), and the sample concentration was diluted to 2 M using three volumes of 50 mM triethylammonium bicarbonate (Sigma-Aldrich, USA). Trypsin-TPCK (Sigma-Aldrich) (1/20 of the total protein amount) was added and gently shaken overnight at 37 °C. Then, the protein was precipitated by 1% TFA (Sigma-Aldrich, USA), and the supernatant was collected in a new tube. The digestion of protein by trypsin was confirmed by SDS-PAGE. The supernatant was desalted using SEP-PAK C18 (Waters, USA), and the purified peptides were then lyophilized at −80 °C. The peptides were treated with a biotin antibody (Abcam, USA) and protein A/G beads (Millipore, USA) to obtain enriched biotinylated peptides. After desalting the solution using the Empore C18 Extraction Disk (3 M USA), the peptides were dissolved in 100 µl of 0.1% TFA and analyzed by mass spectrometry.

### Statistical analysis

The data are expressed as the mean ± SEM. Statistical analysis was performed to analyze the data using SPSS 20.0 (IBM, USA). Fisher’s exact test was used to investigate the relationship between VGLL4 expression and clinicopathological factors. Pearson correlation analysis was used to investigate the correlation between VGLL4 expression and clinicopathological factors. Two factor repeated measures ANOVA was used to analyze the MTT results. Student’s *t*-test was used for the comparison of two means. All experiments were repeated three times independently. Statistical significance was indicated by *P* < 0.05.

## Results

### VGLL4 expression is decreased in TNBC and negatively correlated with Ki67 expression

**Fig. 1:**
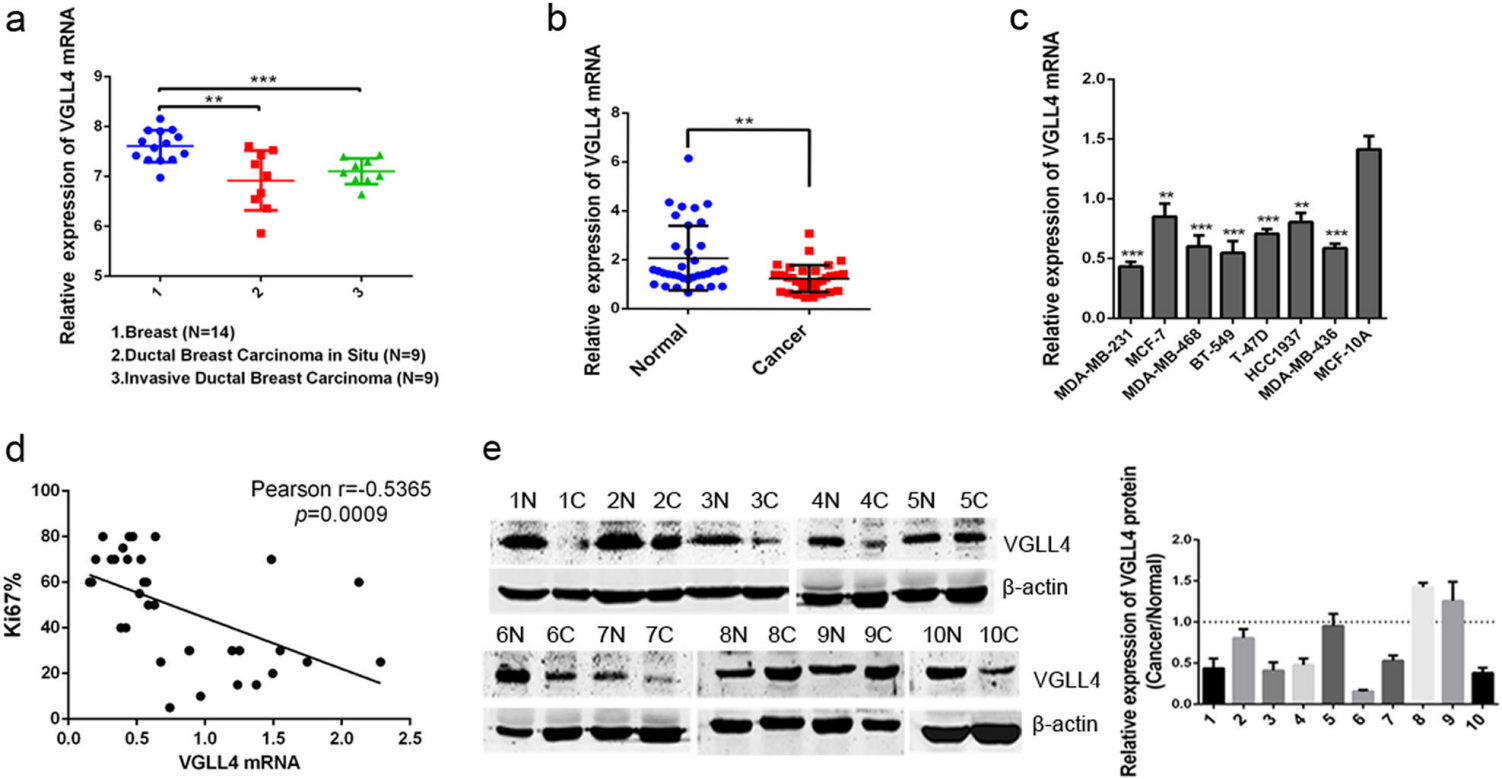
VGLL4 is downregulated in TNBC samples and is negatively correlated with Ki67 expression. **a** VGLL4 expression was lower in breast invasive ductal carcinoma and ductal carcinoma in situ than in normal breast tissue based on the Oncomine database. **b**, **c** The relative expression of VGLL4 mRNA in 35 paired TNBC samples (**b**) and breast cancer cell lines (**c**). **d** The expression of VGLL4 mRNA was negatively correlated with Ki67 expression in TNBC samples. **e** The analysis of VGLL4 protein expression (cancer/normal) in 10 paired TNBC samples. β-actin was used as a loading control. **p < 0.01; ***p < 0.001. All experiments were repeated three times.

According to Oncomine database data (https://www.oncomine.org/), VGLL4 expression was lower in breast invasive ductal carcinoma and ductal carcinoma in situ than in normal breast tissue (Fig. 1a). To examine the expression of VGLL4 in TNBC, we randomly selected tissues from 35 cases of TNBC and paired adjacent normal breast tissues. The results showed that VGLL4 was significantly lower in cancer tissues than in normal tissues (Fig. 1b). In addition, the expression of VGLL4 in breast cancer cell lines (MDA-MB-231, MDA-MB-468, MDA-MB-436, MCF-7, BT-549, T-47D, HCC1937) was lower than that in the human normal breast cell line MCF-10A (Fig. 1c). Meanwhile, we analyzed the correlation between VGLL4 expression and clinical parameters in 35 patients. The results showed that VGLL4 mRNA expression was associated with tumor size and Ki67 expression (Table 1). Correlation analysis showed that VGLL4 mRNA expression was significantly negatively correlated with Ki67 expression (Fig. 1d). Furthermore, we detected the expression of the VGLL4 protein in cancer tissues and adjacent normal tissues from 10 cases randomly selected cases of TNBC and found that the VGLL4 expression in cancer tissues from 7 cases of TNBC (7/10) was lower than that in adjacent normal tissues (Fig. 1e). Based on the above results, VGLL4 expression is downregulated in TNBC and negatively correlated with Ki67 expression.

**Table 1:** Relationship between VGLL4 mRNA expression and clinicopathologic characteristics in TNBC.

Characteristics	No. of cases	VGLL4 mRNA expression		*p* value
		>0.813	<0.813	
Age (year)				0.109
≥50	26	12	14	
<50	9	1	8	
Tumor size (mm)				0.012
≥25	16	2	14	
<25	19	11	8	
Ki67 index				0.032
≥30	27	7	20	
<30	8	6	2	
Histologic grade^a^				0.504
I	1	1	0	
II	8	3	5	
III	26	9	17	
Lymph node metastasis				1.000
Positive	11	4	7	
Negative	24	9	15	
Tumor recurrence				1.000
Yes	5	2	3	
No	30	11	19	

### Downregulation of VGLL4 promotes tumorigenesis in TNBC cells

**Fig. 2:**
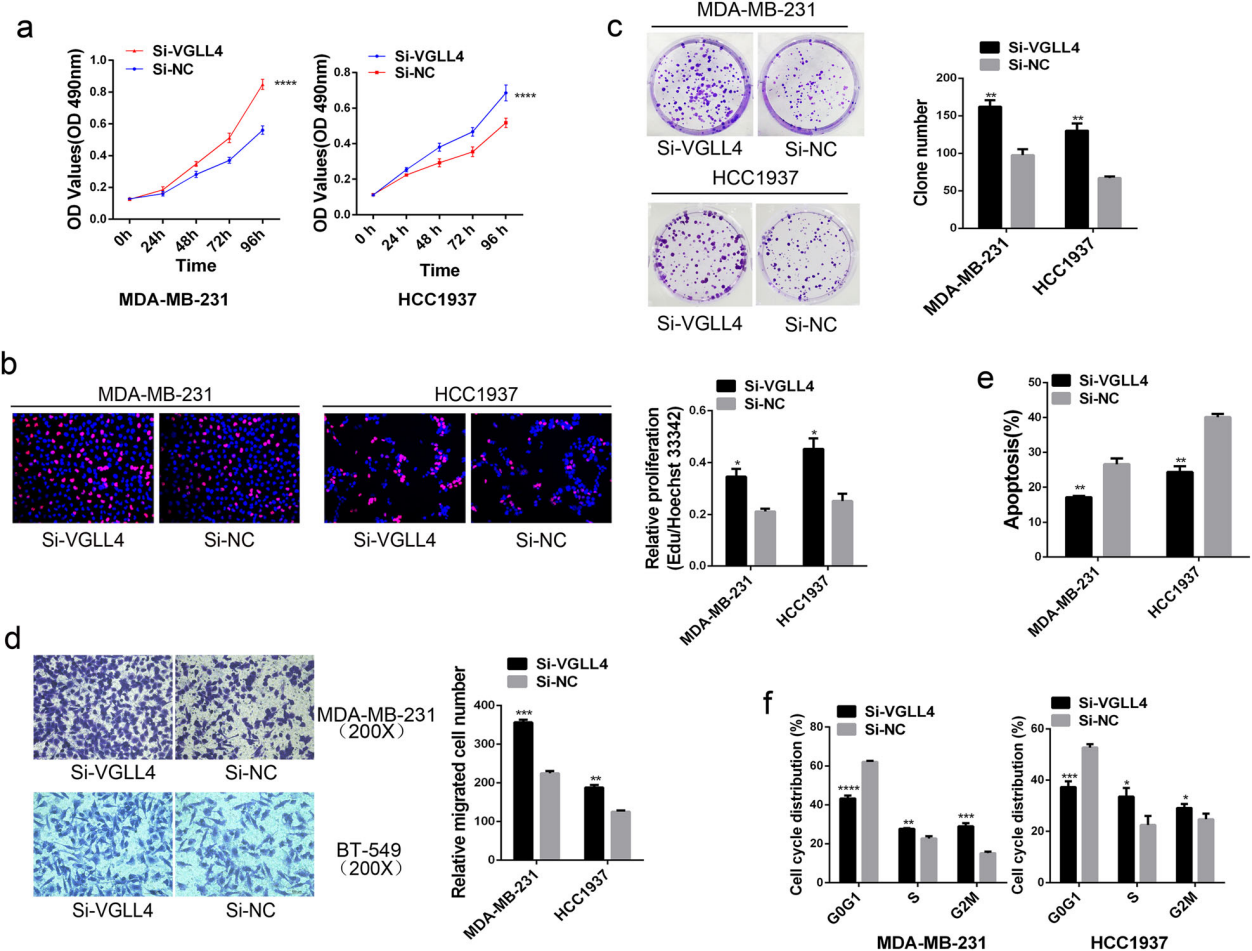
VGLL4 knockdown promotes proliferation and migration, decreases apoptosis and induces a decrease in cell-cycle distribution in the G0G1 phase in TNBC cells. **a**–**c** The viability of breast cancer cells was analyzed by the MTT assay (**a**), the EdU staining proliferation assay (**b**) and a colony formation assay (**c**). **d** Cell migration was measured by a transwell migration assay. **e**, **f** Cell apoptosis (**e**) and cell cycle distribution (**f**) were measured by flow cytometry. Q2 + Q4 represents apoptotic cells (%). *p < 0.05, **p < 0.01, ***p < 0.001, ****p < 0.0001. All experiments were repeated three times.

Given the characteristics of VGLL4 expression in TNBC and its correlation with clinical factors of TNBC, we used the loss and gain of VGLL4 expression to explore the biological function and molecular mechanism of VGLL4 in TNBC cells. As shown in Fig. 2a, b, the downregulation of VGLL4 significantly increased the rate of cell proliferation in both MDA-MB-231 and HCC1937 cells (Fig. 2a, b). Consistently, the downregulation of VGLL4, compared with NC, increased colony formation in both MDA-MB-231 and HCC1937 cell lines (Fig. 2c). Transwell assays revealed that VGLL4 knockdown inhibited cell migration in TNBC cells. These results suggested that VGLL4 knockdown promoted the proliferation and migration of TNBC cells (Fig. 2d). Flow cytometry analysis indicated that the downregulation of VGLL4, compared with NC, decreased apoptosis in MDA-MB-231 and HCC1937 cells (Fig. 2e). In addition, the results of cell cycle analysis showed that the percentage of cells in S and G2/M phase was increased but that the percentage of cells in G0/G1 phase was decreased in MDA-MB-231 and HCC1937 cells in the VGLL4-siRNA group compared with the NC-siRNA group (Fig. 2f). These results indicate that the downregulation of VGLL4 can impact cell-cycle distribution and inhibit apoptosis in TNBC cells.

### VGLL4 overexpression acts as a tumor suppressor in TNBC cells in vitro and in vivo

**Fig. 3:**
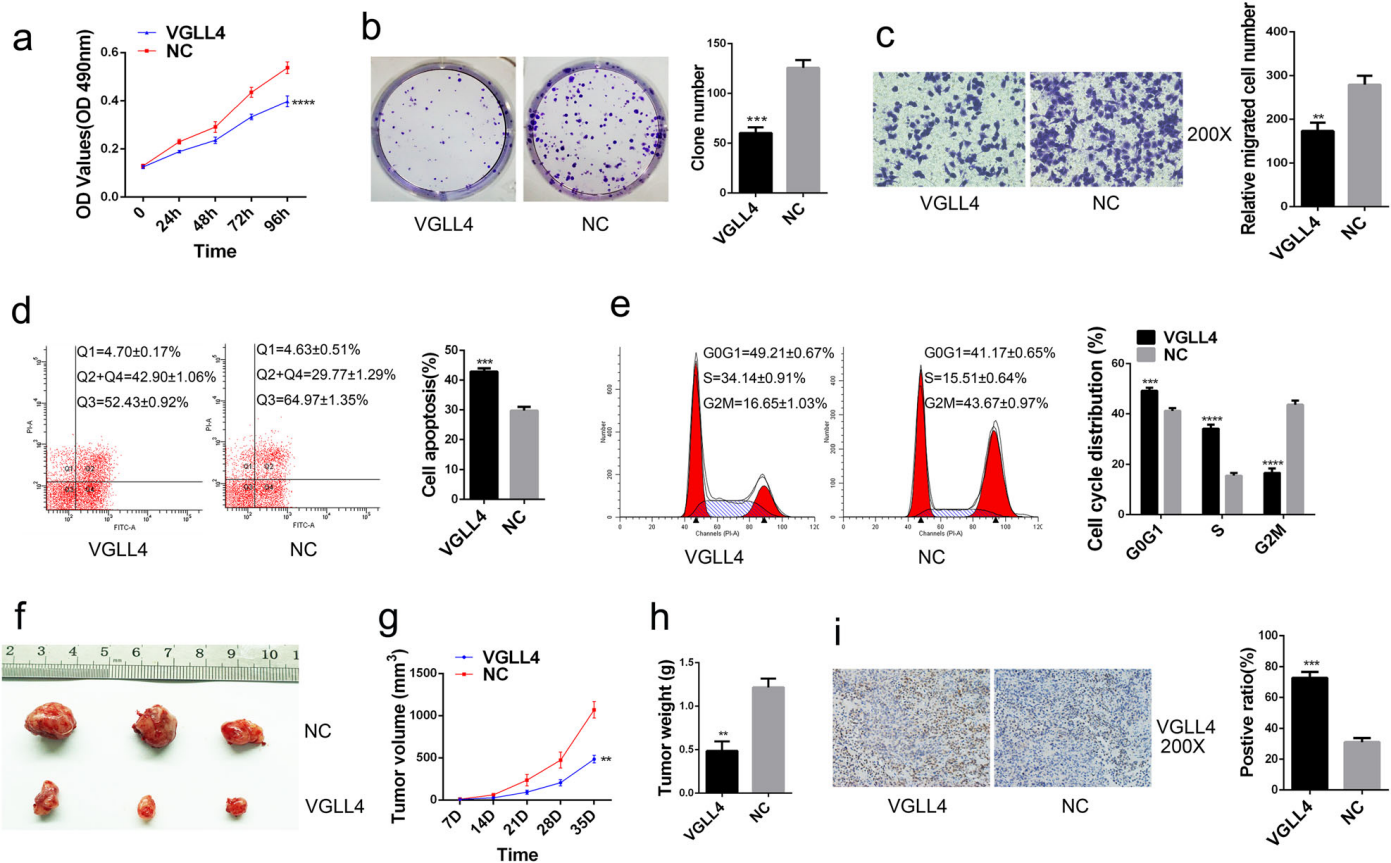
VGLL4 overexpression acts as a tumor suppressor in TNBC cells in vitro and vivo. **a**, **b** Cell viability was determined by the MTT assay (**a**) and a colony formation assay (**b**) after VGLL4 overexpression in TNBC cells. **c** Cell migration was measured by a transwell migration assay. **d**, **e** Cell apoptosis (**d**) and cell-cycle distribution (**e**) were measured by flow cytometry. **f** Tumor formation after inoculation of mice with MDA-MB-231 cells with overexpression of VGLL4 or NC. **g**, **h** The volume (**g**) and weight (**h**) of the xenografts. **i** Representative IHC staining images of VGLL4 protein in the tumors. *p < 0.05, **p < 0.01, ***p < 0.001, ****p < 0.0001. All experiments were repeated three times.

MDA-MB-231 cells with stable overexpression of VGLL4 and its negative control (NC) were used to explore the biological function of VGLL4 in TNBC cells. MTT and colony formation assays showed that VGLL4 overexpression significantly decreased cell proliferation in MDA-MB-231 cells (Fig. 3a, b). The results of the cell migration assay showed that VGLL4 overexpression inhibited cell migration in TNBC cells (Fig. 3c). In addition, VGLL4 overexpression promoted cell apoptosis and arrested the cell cycle in G0G1 phase (Fig. 3d, e). To further assess the effects of VGLL4 in TNBC cells in vivo, MDA-MB-231 cells with stable overexpression and its negative control sequence were expanded and subcutaneously injected into nude mice. The results showed that the growth of the xenograft tumors derived from VGLL4-overexpressing cells was significantly lower than that derived from NC cells (Fig. 3f). In addition, the volume and weight of the tumors in the VGLL4 overexpression group were lower than those of the tumors in the NC group (Fig. 3g, h). IHC staining suggested that VGLL4 expression in tumor sections from the VGLL4 overexpression group was significantly higher than that in tumor sections from the NC group (Fig. 3i). In summary, these findings support the conclusion that VGLL4 serves as a tumor suppressor in TNBC in vitro and vivo.

### Analysis of the potential molecular targets related to TNBC after VGLL4 knockdown or overexpression

**Fig. 4:**
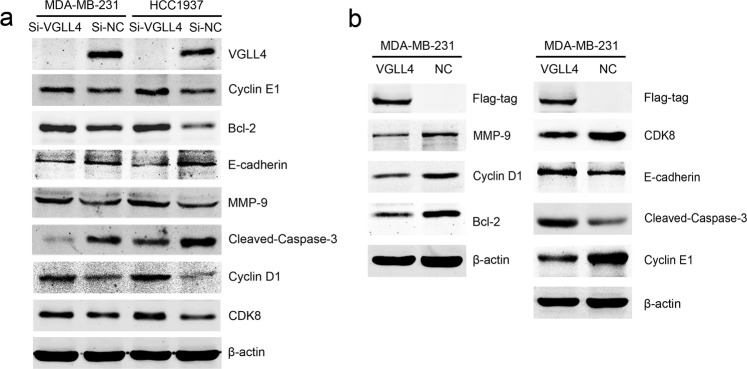
Analysis of the expression of TNBC-related molecular targets after VGLL4 knockdown or overexpression. **a** The expression of Cyclin D1, Cyclin E1, Bcl-2, MMP-9, and CDK8 was upregulated, but the expression of Cleaved Caspase-3 and E-cadherin was downregulated in TNBC cells after VGLL4 knockdown. **b** The expression of Cyclin D1, Cyclin E1, Bcl-2, MMP-9, and CDK8 was downregulated, but the expression of Cleaved Caspase-3 and E-cadherin was upregulated in TNBC cells after VGLL4 overexpression.

To determine the molecular mechanisms of VGLL4 in the progression of TNBC, we tested the expression of biological function-related molecular targets, and we found that VGLL4 knockdown upregulated the expression of Cyclin D1, Cyclin E1, Bcl-2, MMP-9, and CDK8 but downregulated the expression of Cleaved Caspase-3 and E-cadherin in MDA-MB-231 and HCC1937 cells (Fig. 4a). In addition, VGLL4 overexpression reversed the above effects in MDA-MB-231 cells (Fig. 4b). The results indicate that Cyclin D1, Cyclin E1, Bcl-2, MMP-9, and CDK8, Cleaved Caspase-3 and E-cadherin are regulated by VGLL4 and that the changes in these molecules after the downregulation of VGLL4 may exert a promoting effect on the tumorigenesis and progression of TNBC.

### VGLL4 is a direct target of miR-454 in TNBC cells

**Fig. 5:**
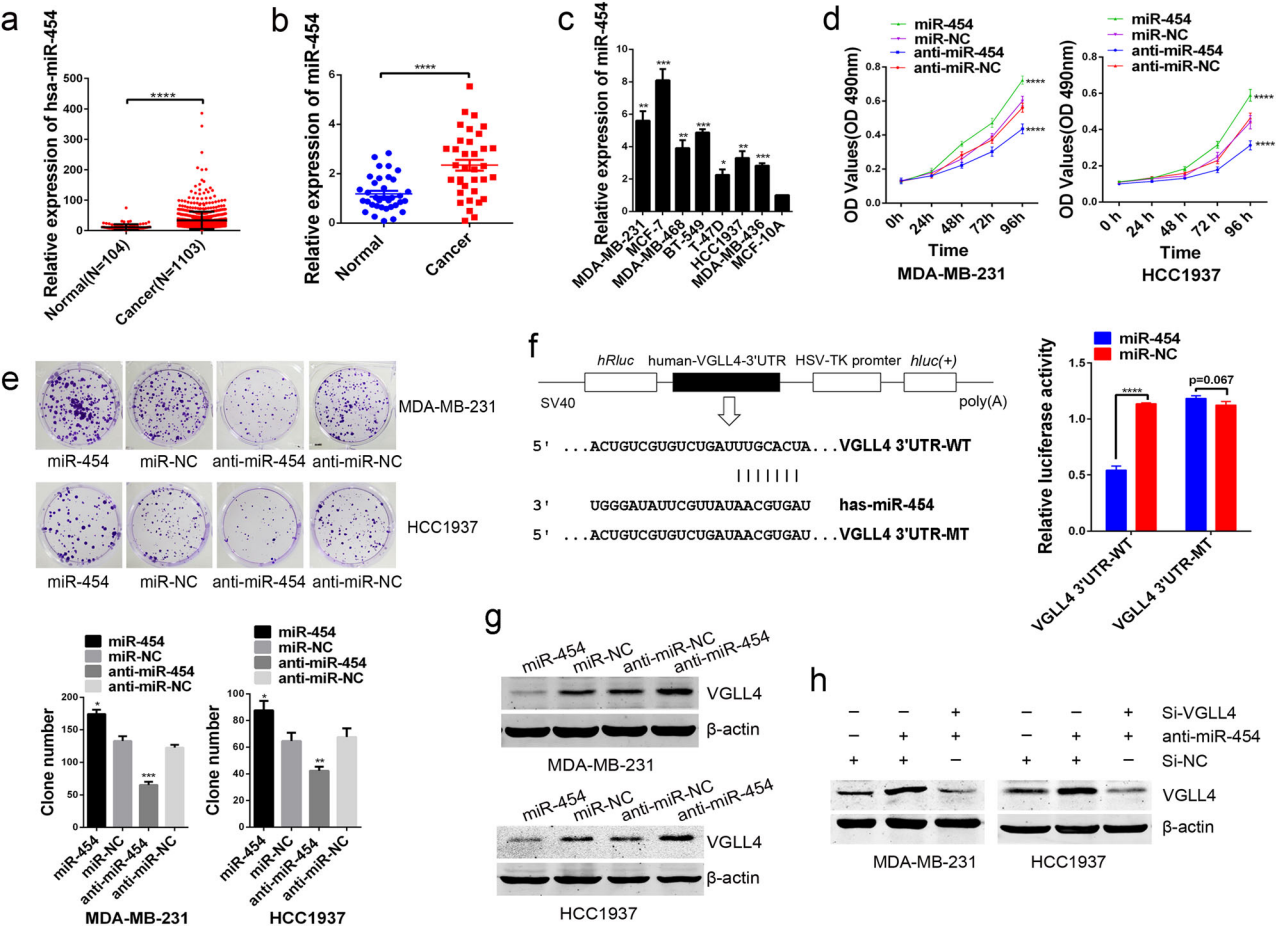
miR-454 promotes the proliferation of TNBC cells by targeting VGLL4. **a** The expression of miR-454 was downregulated in breast cancer samples compared with normal breast tissues based on the TCGA database. **b**, **c** The expression of miR-454 was downregulated in TNBC samples (**b**) and cell lines (**c**). **d**, **e** The viability of TNBC cells was analyzed by the MTT assay (**d**) and a colony formation assay (**e**) after transfection with miR-454 or anti-miR-454. **f** Left panel: A schematic representation of the experimental design used to identify the direct targets of miR-454 in MDA-MB-231 cells. Right panel: A dual-luciferase reporter assay. **g** The analysis of VGLL4 protein expression in TNBC cells after transfection with miR-454 or anti-miR-454. **h** Western blot analysis indicated that anti-miR-454 rescued the downregulation of VGLL4 induced by VGLL4 siRNA at the protein level. *p < 0.05, **p < 0.01, ***p < 0.001, ****p < 0.0001.

We searched databases including TargetScan, miRbase, and miRwalk and found that VGLL4 is a putative target of miR-454. Based on the Cancer Genome Atlas (TCGA) database, miR-454 was shown to be upregulated in breast cancer tissues compared with normal tissues (Fig. 5a). In this study, we found that miR-454 expression was upregulated in TNBC tissues and cell lines when compared with normal tissues and cell lines (Fig. 5b, c). The upregulation of miR-454 significantly increased the proliferation of TNBC cells, and these effects were reversed by inhibition of miR-454 (Fig. 5d, e). To confirm whether VGLL4 is a direct target of miR-454, we constructed plasmids containing the 3′-UTR of wild-type (WT) and mutant-type (MT) of VGLL4, and the luciferase reporter assay found that miR-454 can directly bind to the 3′-UTR of VGLL4 mRNA (Fig. 5f). The upregulation of miR-454 significantly inhibited the expression of the VGLL4 protein, and the expression of the VGLL4 protein was increased following the inhibition of miR-454 (Fig. 5g). In addition, inhibition of miR-454 rescued the downregulation of VGLL4 induced by VGLL4 siRNA at the protein level (Fig. 5h). These findings indicate that miR-454 functions as an oncogene in TNBC at least partly by targeting VGLL4 and that the high expression of miR-454 may be one of the reasons that VGLL4 shows a trend of low expression in TNBC cells.

### The candidate proteins that interact with VGLL4

**Fig. 6:**
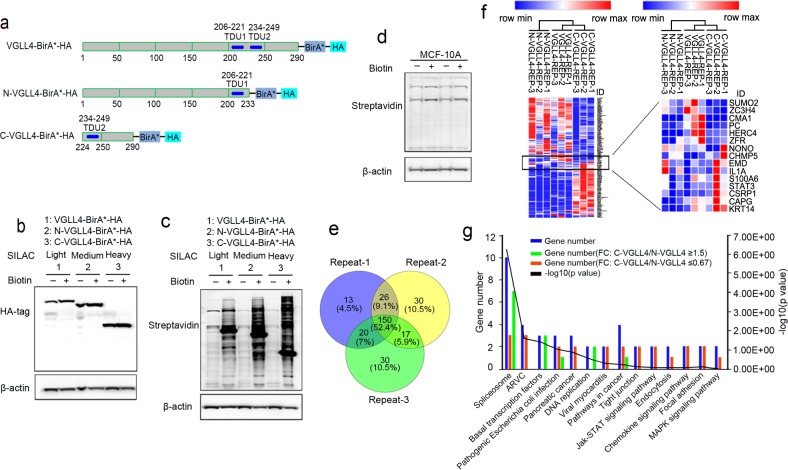
Analysis of candidate proteins that interact with the VGLL4 protein. **a** A schematic representation of the VGLL4-BirA*-HA, N-VGLL4-BirA*-HA, and C-VGLL4-BirA*-HA constructs. **b** The protein expression of VGLL4-BirA*-HA, N-VGLL4-BirA*-HA, and C-VGLL4-BirA*-HA in cells treated with or without biotin. **c** The analysis of biotinylation labeling efficiency in VGLL4-BirA*-HA-, N-VGLL4-BirA*-HA-, and C-VGLL4-BirA*-HA-expressing cells. **d** The analysis of biotinylation labeling efficiency in normal MCF-10A cells. **e** Overlapping sets of biotinylated proteins identified by BioSITe in three replicate experiments. **f** Clustering analysis of candidate proteins is shown by the heatmap. **g** Signaling pathway analysis of candidate proteins by the KEGG pathway database.

Stable VGLL4-BirA*-HA, N-VGLL4-BirA*-HA, and C-VGLL4-BirA*-HA overexpression cells were generated in MCF-10A cell line (Fig. 6a). Some of the samples were used to check corresponding protein expression and the labeling efficiency of biotinylation, and the results show that the corresponding protein expression did not change after treatment with biotin but that biotinylated proteins were significantly increased in the group treated with biotin (Fig. 6b, c). We examined biotinylated protein in normal MCF-10A cells and found that there was no difference upon biotin treatment and no biotin treatment in normal MCF-10A cells (Fig. 6d), which indicates that natural intracellular proteins are rarely biotinylated in the absence of BirA* expression. Then, equal amounts of proteins from the VGLL4-BirA*-HA, N-VGLL4-BirA*-HA, and C-VGLL4-BirA*-HA groups were mixed and tested by BioSITe. The Venn diagram shows that 150 biotinylated proteins overlapped in three replicate experiments (Fig. 6e). These biotinylated proteins accounted for 71.77% (150/209), 67.26% (150/223), and 69.12% (150/217) of the biotinylated proteins in the three replicate groups. The candidate proteins were analyzed by a heatmap and the KEGG pathway database (Fig. 6f, g), and we found that VGLL4-related proteins are involved in several signaling pathways, such as the JAK-STAT pathway and the MAPK signaling pathway.

### VGLL4 suppresses the STAT3 signaling axis by interacting with the STAT3 protein in TNBC cells

**Fig. 7:**
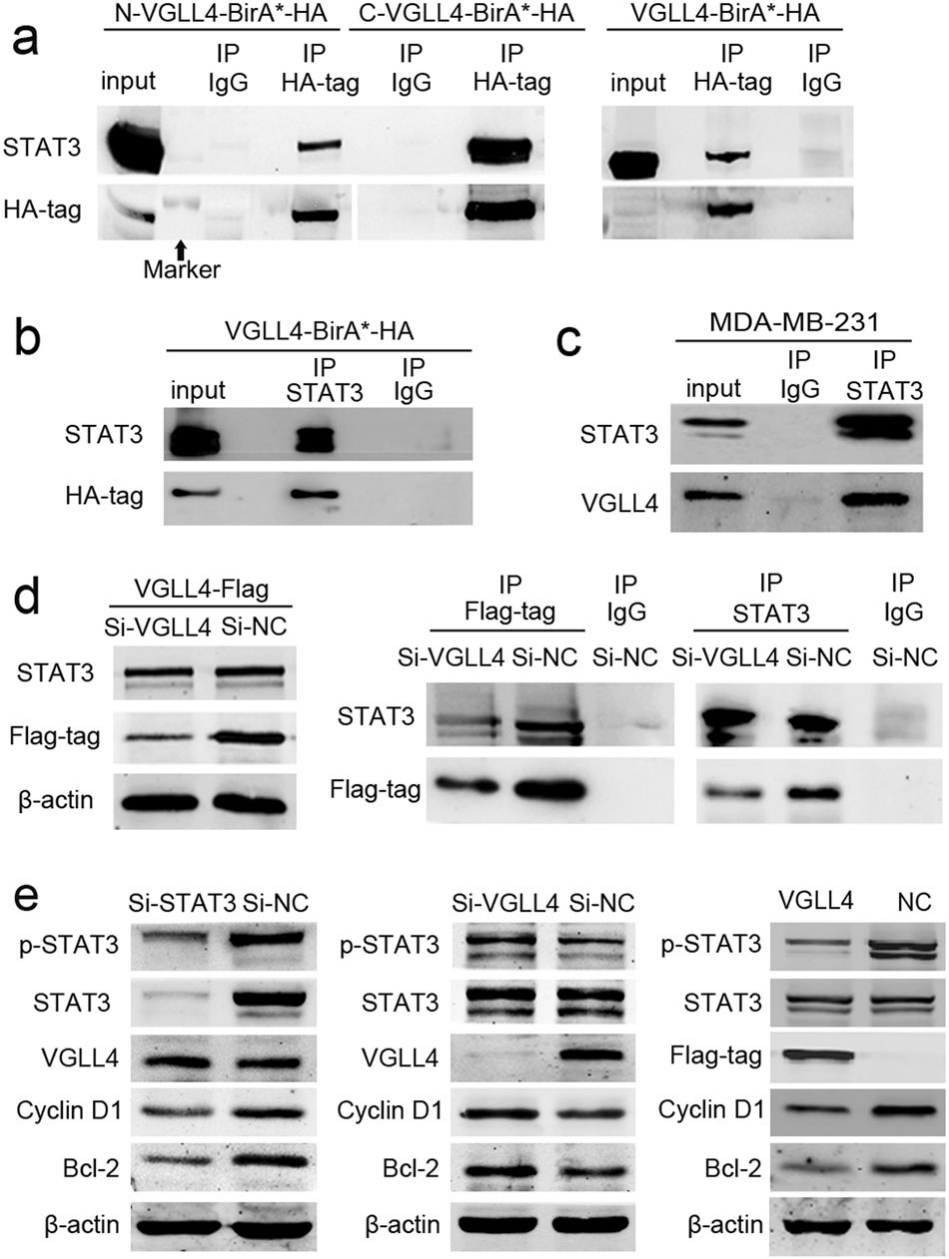
VGLL4 protein suppresses the STAT3 signaling axis by interacting with the STAT3 protein. **a** CoIP assays for STAT3 and HA-tag in MCF-10A cells with VGLL4-BirA*-HA, N-VGLL4-BirA*-HA, and C-VGLL4-BirA*-HA overexpression. **b** CoIP for STAT3 and VGLL4 in MCF-10A cells with VGLL4-BirA*-HA overexpression. **c** CoIP for STAT3 and VGLL4 in normal MDA-MB-231 cells. **d** Left: The expression of exogenous VGLL4 was decreased, but STAT3 expression was unchanged after the transfection of VGLL4 siRNA into VGLL4-Flag-overexpressing MDA-MB-231 cells. Right: CoIP assays showing that the interaction of exogenous VGLL4 with STAT3 was decreased after transfection of VGLL4 siRNA into VGLL4-Flag-overexpressing MDA-MB-231 cells. **e** Left: Western blot analysis of VGLL4 expression in MDA-MB-231 cells after 48 h of transfection with STAT3 siRNA. Middle: Western blot analysis of p-STAT3 and downstream targets of STAT3 in MDA-MB-231 cells after transfection with VGLL4 siRNA. Right: Western blot analysis of STAT3, p-STAT3 and downstream targets of STAT3 in stable VGLL4-Flag-overexpressing MDA-MB-231 cells.

Coimmunoprecipitation (CoIP) was performed using an HA-tag antibody, and STAT3 expression was found in the VGLL4-BirA*-HA, N-VGLL4-BirA*-HA, and C-VGLL4-BirA*-HA groups (Fig. 7a). Therefore, we initially determined that VGLL4 may interact with STAT3. Then, we extracted proteins from MCF-10A cells with VGLL4-BirA*-HA overexpression, and exogenous VGLL4 expression was detected by CoIP using a STAT3 antibody (Fig. 7b). In addition, we extracted proteins from normal MDA-MB-231 cells and found endogenous VGLL4 expression after IP with the STAT3 antibody (Fig. 7c), indicating a protein–protein interaction between VGLL4 and STAT3. More importantly, we found that VGLL4 siRNA significantly downregulated the expression of the exogenous VGLL4 protein in MDA-MB-231 cells overexpressing VGLL4-Flag but did not alter STAT3 protein expression. Given that the VGLL4 antibody could not be used for the IP experiments, we then used a Flag-tag antibody for IP and found that the downregulation of exogenous VGLL4 protein expression decreased the exogenous VGLL4 protein binding to the STAT3 protein. Similarly, the expression of the exogenous VGLL4 protein was decreased in the VGLL4-siRNA group compared with the NC group after IP with the STAT3 antibody (Fig. 7d). The above results indicate that the interaction between exogenous VGLL4 and the STAT3 protein can be decreased by downregulating the expression of exogenous VGLL4.

To investigate whether there is a correlation between VGLL4 expression level and STAT3 signaling pathway activation in TNBC cell lines, we measured the expression of VGLL4, p-STAT3 and downstream targets of the STAT3 signaling pathway, such as Cyclin D1 and Bcl-2, in TNBC cell lines (MDA-MB-231, MDA-MB-436, BT-549, and MDA-MB-468) and normal mammary epithelial MCF-10A cells. The results showed that VGLL4 expression was significantly lower in MCF10A cells than in TNBC cells, and importantly, VGLL4 expression was significantly lower in MDA-MB-231 cells than in other TNBC cells (MDA-MB-436, BT-549, and MDA-MB-468 cells). The expression of p-STAT3 and downstream targets of the STAT3 signaling pathway, Cyclin D1 and Bcl-2 expression in TNBC cell lines were significantly higher than that in MCF10A cells. The results indicate a negative correlation between VGLL4 expression and STAT3 signaling pathway activation in TNBC cell lines (Sup Fig. 1).

To explore the regulatory relationship between VGLL4 and STAT3, we first investigated whether STAT3 expression changes can affect VGLL4 expression. Using STAT3 siRNA to downregulate STAT3 expression, it was found that phosphorylated STAT3 and total STAT3 expression was downregulated compared with that in the NC group, while the expression of the STAT3 downstream genes, such as the protein expression of Bcl-2 and Cyclin D1 were both downregulated; however, there was no change in VGLL4 protein expression, indicating that expression changes in STAT3 did not affect VGLL4 protein expression levels. Furthermore, in MDA-MB-231 cells, we used VGLL4 siRNA to downregulate VGLL4 expression and detected phospho-STAT3 expression, total STAT3 expression and the protein expression of downstream targets of STAT3. The results showed that, compared with that in the si-NC group, the expression of the phospho-STAT3 protein was downregulated, but total STAT3 protein expression was not changed after VGLL4 downregulation. Regarding the downstream targets of STAT3, the protein expression of Bcl-2 and Cyclin D1 was higher in the VGLL4 siRNA group than in the NC-siRNA group. At the same time, in VGLL4-overexpressing MDA-MB-231 cells, we found that the expression of the phospho-STAT3 protein was downregulated, the protein expression of Bcl-2 and Cyclin D1 was decreased, but the expression of total STAT3 protein was not altered (Fig. 7e). Taken together, the above experiments indicate that the VGLL4 protein may inhibit STAT3 phosphorylation and its downstream transcriptional activity by interacting with the STAT3 protein.

## Discussion

Targeted therapies, such as the mTOR inhibitor everolimus^22^, the latest CDK4/6 inhibitor, palbociclib^23^, for hormone receptor-positive breast cancer, and trastuzumab^24^ for HER2-positive breast cancer, have made certain advancements in the treatment of breast cancer. However, there are no effective targets for patients with TNBC in clinical practice, and exploring useful molecular targets is important for the treatment of TNBC.

Our current study determined that VGLL4 was downregulated at both the mRNA and protein levels in TNBC. Ki67 is a biomarker associated with tumor proliferation, and its high expression suggests strong tumor proliferative capacity^25^. Further analysis found a significant negative correlation between VGLL4 mRNA expression and Ki67 expression. This suggests that low expression of VGLL4 may be beneficial for the proliferation of TNBC cells. Our data showed that the downregulation of VGLL4 in TNBC cells promoted cell proliferation and migration and reduced apoptosis. Regarding the cell cycle, VGLL4 knockdown decreased the proportion of cells in the G0G1 phase but increased the proportion of cells in the S phase and G2M phase. However, overexpression of VGLL4 reversed the above biological functions of TNBC cells. Furthermore, in animal experiments, overexpression of VGLL4 inhibited the growth of TNBC cells in vivo, and the tumor volume and weight in the VGLL4 overexpression group was decreased significantly compared with those in the NC group. The IHC results from the tumors showed that VGLL4 expression was significantly higher in the VGLL4 overexpression group than in the NC group, indicating that VGLL4 overexpression may be the cause of inhibition of tumor growth in vivo in mice.

Bioinformatics prediction revealed that miR-454 may target VGLL4 for binding. It has been reported that miR-454 is differentially expressed in colon cancer^26^, renal cancer^27^, and esophageal cancer^28^. In these cancers, overexpression of miR-454 can promote the proliferation of colon cancer cells^26^. High expression of miR-454 in patients with triple-negative breast cancer indicates lower disease-free survival and overall survival than that indicated by low expression of miR-454. Meanwhile, high expression of miR-454 indicates a poor prognosis in TNBC^29^. The results confirmed that overexpression of miR-454 significantly promoted proliferation. The downregulation of miR-454 expression reverses the above biological functions, and thus, the results demonstrate that miR-454 may promote tumorigenesis in TNBC. Importantly, our data demonstrated that miR-454 can directly bind to the 3′-UTR of VGLL4 mRNA. In addition, miR-454 overexpression inhibited the expression of the VGLL4 protein in TNBC cells, while the VGLL4 protein was upregulated after inhibition of miR-454. Thus, miR-454 may downregulate VGLL4 expression by blocking its translation. High expression of miR-454 may be one of the causes of low VGLL4 expression in TNBC samples. The miR-454-VGLL4 axis may provide a new direction for clinical diagnosis and targeted therapy for TNBC.

Complex cell functions are usually the result of the synergistic effect of multiple proteins affecting molecular assembly and signal transduction. Further investigation revealed that the downregulation of VGLL4 upregulated the expression of apoptosis-related Bcl-2 and the cell-cycle regulators Cyclin D1, Cyclin E1, and CDK8, and the upregulation of these molecules was conducive to tumor proliferation. In addition, the expression of the migration-associated protein MMP-9 was found to be upregulated after the downregulation of VGLL4, but the expression of Cleaved Caspase-3 and E-cadherin was downregulated. VGLL4 may be involved in the development of cancer through a variety of signaling pathways. It has been reported that overexpression of VGLL4 in gastric cancer can increase the expression level of E-cadherin and decrease the expression level of β-catenin, indicating that VGLL4 inhibits epithelial-mesenchymal transition (EMT) in gastric cancer cells in part by negatively regulating the Wnt/β-catenin signaling pathway^30^. CDK8 can directly or indirectly regulate β-catenin the activation of transcriptional activity^31^. β-catenin is a key component of the Wnt/β-catenin signaling pathway, and VGLL4 may participate in the Wnt/β-catenin signaling pathway by regulating CDK8 expression.

To explore the proteins that may interact with VGLL4, we used SILAC in combination with BioSITe. The results showed that candidate proteins are associated with multiple cancer signaling pathways, such as pathways in cancers, the JAK-STAT pathway, and the MAPK signaling pathway. The JAK-STAT pathway plays an important role in the development of many malignancies^32^, and STAT3, as a key regulator of the JAK-STAT3 signaling pathway, can promote cell proliferation, survival, migration, invasion, and immunosuppression in tumors including breast cancer^33–35^. The STAT3 inhibitor pyrimethamine displays anticancer and immune stimulatory effects in murine models of breast cancer^36^. It has also been reported that STAT3 plays an important role in the hypoxia-induced chemoresistance of MDA-MB-231 cells, a triple-negative breast cancer cell line^37^. Further analysis revealed that the STAT3 protein was expressed in HA-tag antibody immunoprecipitated protein from VGLL4-BirA*-HA-, N-VGLL4-BirA*-HA- and C-VGLL4-BirA*-HA-expressing cells, respectively. This suggests that VGLL4 may interact with STAT3, and further CoIP experiments confirmed that VGLL4 has a protein–protein interaction with STAT3. It has been reported that MMP2, MMP-9, Cyclin D1, Cyclin E1, and Bcl-2 are downstream of STAT3 transcriptional regulation^38–40^, and STAT3 is primarily activated by its protein phosphorylation and then activates downstream transcription^33^. By inhibiting and overexpressing VGLL4, we found that phospho-STAT3, Cyclin D1 and Bcl-2, downstream targets of STAT3, were elevated and inhibited, respectively. In addition, STAT3 knockdown significantly downregulated STAT3 phosphorylation and its downstream transcription but did not affect VGLL4 protein expression. The above results indicate that VGLL4 may decrease STAT3 activity by interacting with STAT3 in TNBC cells.

In summary, the expression of VGLL4 is low in TNBC samples and is negatively correlated with Ki67. By silencing and overexpressing VGLL4, we demonstrated that VGLL4 is a critical tumor suppressor of TNBC cells. Further studies indicated that miR-454 acts as an oncogenic role in tumorigenesis of TNBC by targeting VGLL4. Importantly, this study showed that the VGLL4-mediated regulatory mechanism of STAT3 might provide a mechanistic insight into the biological behavior and therapeutic targets of TNBC.

## Supplementary information

Supplementary Information
