# Supplementary material for: VGLL4 interacts with STAT3 to function as a tumor suppressor in triple-negative breast cancer
Source: Exp Mol Med. 2019 Nov 20;51(11):141. doi: 10.1038/s12276-019-0338-8 (PMC6868227; doi:10.1038/s12276-019-0338-8)
Supplement: Supplementary file 1 — Supplementary Information [file 12276_2019_338_MOESM1_ESM.docx]

**
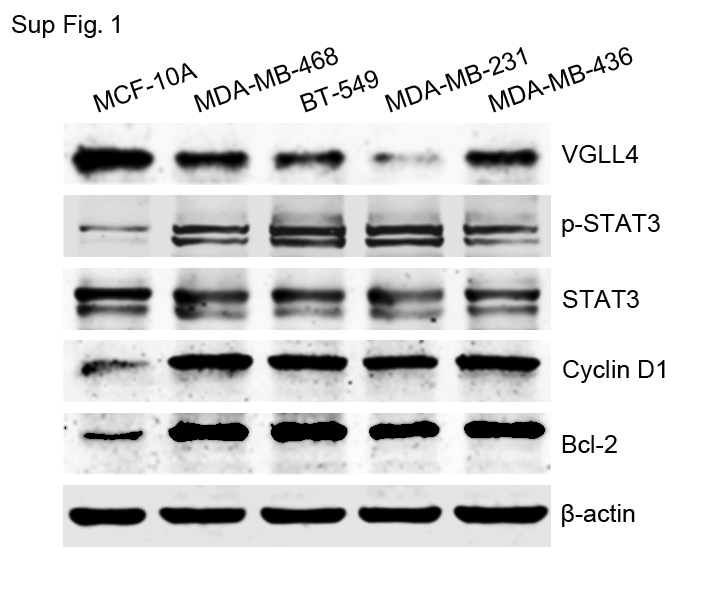
**

**Supplementary figure legends**

**Supplementary Figure 1. VGLL4 expression level is negatively correlated with STAT3 signaling pathway activation in TNBC cell lines.** VGLL4 expression was significantly lower in MCF10A cells than in TNBC cells (MDA-MB-231, MDA-MB-436, BT-549, and MDA-MB-468 cells), but the expression of p-STAT3, downstream targets of the STAT3 signaling pathway, Cyclin D1 and Bcl-2 in TNBC cell lines were significantly higher than that in MCF10A cells.

Supplementary Table 1. Essential information of synthetic sequences and primers used in this study.

| Name | | Sequence |
| --- | --- | --- |
| VGLL4 | Primer-Forward | 5'-AACTGCAACCTCTCGCACTG-3' |
|  | Primer- Reverse | 5'-GCTCGGGCTCCTTGTAATTCT-3' |
| β-actin | Primer- Forward | 5'-CAGAGCCTCGCCTTTGCC-3' |
|  | Primer- Reverse | 5'-GTCGCCCACATAGGA ATC-3' |
| has-miR-454 | Primer-RT | 5'-GTCGTATCCAGTGCGTGTCGTGGAGTCGGCAATTGCACTGGATACGACACCCTA-3' |
|  | Primer- Forward | 5'-TAGTGCAATATTGCTTATAGGGT-3' |
|  | Primer- Reverse | 5'-CAGTGCGTGTCGTGGAGT -3' |
| U6 | Primer-RT | 5'-GTCCTATCCAGTGCAGGGTCCGAGGTGCACTGGATACGACAAAATATGGAAC-3' |
|  | Primer- Forward | 5'-TGCGGGTGCTCGCTTTCGCAGC-3' |
|  | Primer- Reverse | 5'-CCAGTGCAGG GTCCGAGGT-3' |
| CTGF | Primer- Forward | 5’- ACCGACTGGAAGACACGTTTG-3’ |
|  | Primer- Reverse | 5’ -CCAGGTCAGCTTCGCAAGG-3’ |
| CYR61 | Primer- Forward | 5’ -CAGGACTGTGAAGATGCGGT-3’ |
|  | Primer- Reverse | 5’ -AGCCTGTAGAAGGGAAACGC-3’ |
| PTGS2 | Primer- Forward | 5’- GTTCCACCCGCAGTACAGAA-3’ |
|  | Primer- Reverse | 5’ -AGGGCTTCAGCATAAAGCGT-3’ |
| VGLL4 Si-RNA | | Sense: 5’ -CCACGUCUCCAAAAUGAGUTT- 3’  Antisense: 5’ -ACUCAUUUUGGAGACGUGGTT- 3’ |
| STAT3 Si-RNA | | Sense: 5’- GGGACCUGGUGUGAAUUAUTT- 3’  Antisense: 5’ -AUAAUUCACACCAGGUCCCTT -3’ |
| Si-RNA Negative control | | Sense: 5’ -UUCUCCGAACGUGUCACGUTT-3’  Antisense: 5’-ACGUGACACGUUCGGAGAATT-3’ |
| hsa-miR-454 mimics | | Sense: 5’ -UAGUGCAAUAUUGCUUAUAGGGU -3’  Antisense: 5’-ACCCUAUAAGCAAUAUUCGACUA- 3’ |
| microRNA mimics NC | | Sense: 5’-UUUGUACUACACAAAAGUACUG-3’，  Antisense: 5’-CAGUACUUUUGUGUAGUACAAA-3’ |
| hsa-miR-454 inhibitors | | 5’ -ACCCUAUAAGCAAUAUUCGACUA- 3’ |
| microRNA inhibitor NC | | 5’-CAGUACUUUUGUGUAGUACAAA-3’ |
| VGLL4- Forward-BamH1 | | 5’-CCTTCTCTAGGCGCCGGCCGGGATCCACCATGGAGACGCCATTGGATGTTTTG-3’ |
| VGLL4- Reverse -XHO1 | | 5’-CTTTGTAGTCGGATCCGGAGACCACAGAGGGGGA-3’ |
| N-VGLL4- Forward -BamH1 | | 5’-CCTTCTCTAGGCGCCGGCCGGGATCCACCATGGAGACGCCATTGGATGTTTTG-3’ |
| N-VGLL4- Reverse -XHO1 | | 5’-CGGTGTTGTCCTTCAATTGCTCGAGGATGGACACGGAGTTGGGTGCC-3’ |
| C-VGLL4- Forward -BamH1 | | 5’-CTTCTCTAGGCGCCGGCCGGGATCCACCATGGAGCCGGCACCCAACTCC-3’ |
| C-VGLL4- Reverse -XHO1 | | 5’-CGGTGTTGTCCTTCAATTGCTCGAGGGAGACCACAGAGGGGGAGTGAC-3’ |
